# Changes in Respiratory Pathogens before and after the COVID-19 Pandemic (2018–2021)

**DOI:** 10.1155/2022/1324052

**Published:** 2022-10-10

**Authors:** Ki Yeon Kim, Jae Soo Kim, Young Ki Lee, Ga Yeon Kim, Bo Kyeung Jung

**Affiliations:** ^1^Department of Biomedical Laboratory Science, College of Health Sciences, Dankook University, Cheonan, Chungnam, Republic of Korea; ^2^Department of Laboratory Medicine, Dankook University Hospital, Cheonan, Chungnam, Republic of Korea; ^3^Department of Public Health, Dankook University Graduate School, Cheonan, Chungnam, Republic of Korea; ^4^Department of Laboratory Medicine, Dankook University College of Medicine, Cheonan, Chungnam, Republic of Korea

## Abstract

**Objective:**

This study is aimed at investigating the pattern of change occurring in respiratory pathogens before and after the outbreak of COVID-19, a type of viral pneumonia for which a pandemic was declared (March 2020). The results were analyzed by gender and age to identify the association between personal hygiene and prevention of infection by respiratory pathogens.

**Methods:**

A retrospective analysis was performed on 39,814 sputum, bronchial aspirate, and transtracheal aspirate samples obtained from 15,398 patients visiting a university hospital, located in Chungcheongnam-do, South Korea, between January 2018 and December 2021. From 4,454 patients whose samples were culture positive for bacteria, 6,389 strains were isolated and further cultured.

**Results:**

The mean age of the outpatients with respiratory pathogens was 66.2 years, and the comparison of the culture test results by gender showed that 64.9% (2,892/4,454) were male and 35.1% (1,562/4,454) were female. Compared to the pre-COVID-19 pandemic period, the number of outpatients with a request for respiratory microbial cultures after the onset of the pandemic was reduced by 20.7% and the number of outpatients with a positive culture result was reduced by 23.0%. The number of respiratory samples received was reduced by 6.7% after the pandemic, while the sample positive rate was reduced by 18.3%. Among the isolated microbial strains, there was a significant decrease of 43.1% for the *Acinetobacter baumannii* complex, 60.5% for *Streptococcus pneumoniae*, 67.2% for *Haemophilus influenzae*, and 78.1% for *Moraxella catarrhalis* when compared with pre-COVID-19 levels. The distribution of respiratory microbial strains by age group showed that the highest percentage of isolated strains was in patients in their 70s.

**Conclusions:**

The improvements in personal hygiene due to the COVID-19 pandemic exerted a substantial influence on the pattern of change in other common respiratory microorganisms, which highlights the importance of personal hygiene management in the prevention of respiratory infections.

## 1. Introduction

Pneumonia is known to have a high incidence rate, predominantly in developing countries [1]. Recently, however, mortality associated with pneumonia has increased in advanced countries including South Korea. Pneumonia has consequently become the 3^rd^ most common cause of death in South Korea, and notably, it is the number one cause of death due to infectious diseases. Cause of death comparisons by year in South Korea for 2010–2020 show that pneumonia has increased from 6^th^ in 2010 to 3^rd^ most common in 2020 [2]. Pneumonia is a bacterial, viral, or fungal infection of the lungs that has the potential to cause a pandemic [3]. The Coronavirus Disease-19 (COVID-19) is a type of pneumonia caused by a viral infection. It leads to respiratory infection and was first reported to the World Health Organization in December 2019, and a pandemic was declared on March 11, 2020 [4]. The infectivity of pneumonia is considered to be high as it accompanies fever, sputum, chest pain, labored breathing, and cough [5]. On January 20, 2020, the first confirmed COVID-19 patient was reported in South Korea [6], and as of April 17, 2022, the total cumulative number of confirmed COVID-19 patients is 16,305,752 [7]. The WHO is continuing to conduct various disinfection policies which are aimed at reducing the global spread of COVID-19.

For the management of infectious diseases, prevention clearly precedes treatment. The world has relied heavily on a strategy of nonpharmaceutical intervention (NPI) to prevent the spread of COVID-19 [8]. The current disease prevention authorities in South Korea emphasize personal hygiene management such as mask-wearing and hand washing as well as social distancing and recommend that people should comply with public preventive measures to block the infection and contagion of COVID-19. Through the operation of the Central Disaster Management Headquarters at the Korea Centers for Disease Control and Prevention (KCDC), government-wide efforts have been focused on the prevention of epidemics [9]. Respiratory pathogens infect the nasal mucosa through hands contaminated with respiratory or intestinal viruses and various nonviral pathogens [10–12]. Viral and bacterial pathogens present on hands can be removed by washing with soap [13, 14]. This practice has consequently been recommended as one of the most important measures by which to prevent the spread of viruses including SARS-CoV-2 [15], and the current pandemic has led to a shift in the beliefs and behaviors regarding hand hygiene across the general public. Mask-wearing has also been shown to be effective in the prevention of disease for both healthy and asymptomatic individuals [16]. The practice of mask-wearing is critical as it helps to prevent droplets or airborne transmission that could cause mass infection [17]. At present, mask-wearing is recommended as the most effective way by which to block the spread of COVID-19 in a pandemic situation [18]. Mask-wearing by health professionals and the general public has been shown to decrease the risk of respiratory viral infections [19], and mask-wearing in public places and multiuse facilities has been proven to be an effective strategy by which to prevent COVID-19 infections [20]. In addition, public mask-wearing has been shown to be a valuable method by which to reduce community transmission and mortality, to ultimately reduce the burden of infectious diseases on healthcare systems [16]. For example, mask-wearing by community residents was found to contribute to the control of COVID-19 by lowering the rate of asymptomatic infection [21]. A meta-analysis on the effects of mask-wearing showed that mask-wearing could reduce the rate of COVID-19 infection by 95% [22]. In South Korea, the revised infection protection act has imposed fines on noncompliance with mask-wearing regulations since October 2020 as an effort to increase the practice [23].

As can be seen, the NPI strategies including hand hygiene, mask-wearing, and social distancing have been reported to assist in reducing the rate of COVID-19 infection by 30%–70% depending on the study site, severity of the infectious disease, and applied quarantine measures [24, 25]. It is also possible that the NPI strategies may influence the onset and spread of other diseases caused by respiratory pathogens as well as COVID-19.

This study investigated the pattern of change in respiratory microorganisms across outpatients at a hospital before and after the COVID-19 pandemic to determine the effects of the changes in our personal hygiene practices such as mask-wearing and hand washing, on the type and frequency of microorganisms causing respiratory infections through comparison before and after the COVID-19 pandemic.

In this study, the isolated microorganisms of respiratory samples from patients visiting an 850-bed university hospital in Chungcheongnam-do, South Korea, were retrospectively analyzed using data from 2018 and 2019 before the COVID-19 outbreak and 2020 and 2021 after the COVID-19 outbreak. The aim was to determine the pattern of change in respiratory microorganisms and the frequency of isolated microorganisms according to the patient age and gender, year, and type of microorganism so as to identify the association between personal hygiene and the prevention of the spread of respiratory pathogens.

## 2. Materials and Methods

### 2.1. Subjects

This study analyzed 39,814 respiratory samples from 15,398 outpatients at an 850-bed university hospital located in Chungcheongnam-do, South Korea, that were submitted for bacterial culture during the period between January 2018 and December 2021. Respiratory samples included sputum, bronchial aspirates, and transtracheal aspirates. Bacterial growth in cultures was identified for 4,454 samples, from which 6,389 strains were isolated. The median age of the patients was 66.2 years.

This study was approved by the Institutional Review Board at Dankook University Hospital (IRB No. 2022-01-008). Data from the patient electronic medical records (EMRs) were analyzed retrospectively. The requirement for informed consent was waived because this study was based on a retrospective analysis of EMRs.

### 2.2. Methods

The submitted respiratory samples were spread on blood agar plates and MacConkey agar and cultured in a 5% CO_2_ incubator at 36°C for 18–24 h. For the detection of *Haemophilus* spp., *Staphylococcus aureus* was streaked on the blood agar plate as a test for satellitism. Each colony of the cultured bacteria was applied to 0.45% PBS McFarland 0.5 for identification using VITEK 2 GP, GN, NH, and YST ID cards (BioMerieux, Durham, USA) to test for Gram-positive bacteria, Gram-negative bacteria, *Neisseria*-*Haemophilus*, and yeast, respectively.

### 2.3. Data Collection and Statistical Analysis

Patient data were collected through retrospective analysis of the EMR. For statistical analyses, Microsoft Excel (Microsoft, Redmond, WA, USA) was used. The median age of the patients was calculated using a pivot table. The age was also calculated by dividing the number of living days by 365 and rounding off to the nearest tenth. If an identical strain was cultured multiple times from the same patient in the same year during the study period, the first result was used, while if an identical strain was cultured from the same patient in a subsequent year, the result was included again. If more than one strain was isolated, all strains were included in the result. The level of significance was set to *p* < 0.05. The Poisson test was used for proportion analysis.

## 3. Results

### 3.1. Isolation Frequency by Age and Gender

The proportion of positive bacterial cultures from 2018–2021 was greatest for those in their 70s at 27.2%, followed by those in their 80s, 60s, and 50s (Table 1). The detection rate of respiratory microorganisms by age showed 41 (0.9%) children aged ≤ 9 years, 36 (0.8%) adolescents, and 4,377 (98.3%) adults aged ≥ 19 years, among whom the older adults aged ≥ 60 years accounted for the majority at 3,294 (74.0%). The gender distribution showed that the number of men (*n* = 2,892; 64.9%) exceeded women (35.1%) by 29.9% and there was a ratio of 1.85 : 1 (male : female). From adolescence to their 70s, the number of male patients was significantly higher, but the gap narrowed in their 80s, and there were more female patients in their 90s.

### 3.2. Comparison of Outpatient Respiratory Samples by Year

The total number of outpatients was 15,398 from 2018–2021, however, the number decreased by 20.7% from 8,588 before the COVID-19 pandemic to 6,810 after the COVID-19 pandemic. The number of patients with a culture positive for pathogens was 4,454, showing a decrease of 23.0% from 2,516 before the COVID-19 pandemic to 1,938 after the onset of the COVID-19 pandemic (Table 2).

The total number of received respiratory samples for 2018–2021 was 39,814. As there were 9,083 respiratory samples in 2017, the rate of increase was 13.4% before the COVID-19 pandemic within the study period, but the rate dropped by 6.7% after the onset of the COVID-19 pandemic.

The sample positive rate was 33.6% and 34.9% in 2018 and 2019, respectively, before the onset of the COVID-19 pandemic, followed by 29.3% and 30.6% in 2020 and 2021, respectively, after the onset of the COVID-19 pandemic. The sample positive rate decreased by 18.3% after the onset of the COVID-19 pandemic.

After statistical analysis using the Poisson test, the number of patients, number of samples, and number of positive samples significantly decreased (*p* < 0.05) both after the onset of the COVID-19 pandemic (2018, 2019 vs. 2020, 2021). However, the number of positive patients did not significantly decrease after the declaration of the COVID-19 pandemic (*p* > 0.05) (Table 2).

### 3.3. Changes in the Pathogen Isolation Frequency by Year

**Figure 1:**
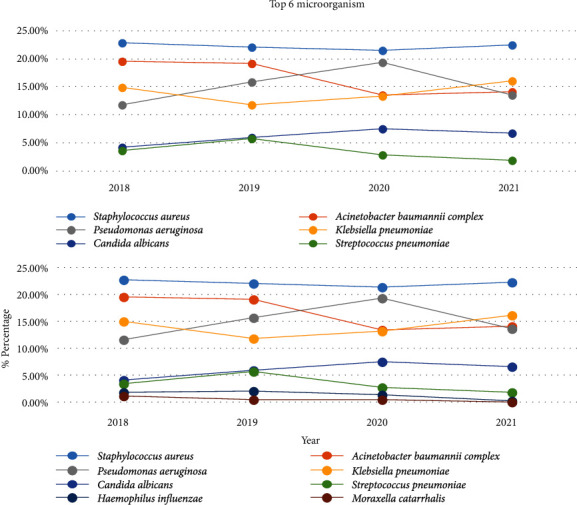
Isolation frequency of major respiratory pathogens by year.

The sputum culture test was performed on 39,814 samples in total from 2018–2021. Positive cultures were found for 12,823 (32.2%) samples and with the exclusion of duplicates, 6,389 strains of respiratory microorganisms were isolated. The number of isolated microorganisms during the study period showed variations according to strain, and a significant fall was observed in the distribution of the *Acinetobacter baumannii* complex, *Streptococcus pneumoniae*, *Haemophilus influenzae,* and *Moraxella catarrhalis* (Table 3). The most frequently cultured pathogen in the 6,389 pathogenic strains was *S. aureus* (22.2%; 1,420/6,389), followed by the *A. baumannii* complex (16.9%; 1,078/6,389), *Pseudomonas aeruginosa* (14.9%; 954/6,389), *Klebsiella pneumoniae* (14.0%; 895/6,389), *Candida albicans* (6.0%; 383/6,389), and *S. pneumoniae* (3.7%; 233/6,389) (Figure 1).

The comparison of the isolation frequency of the bacteria before and after the onset of the COVID-19 pandemic showed that the mean frequency in 2018 and 2019, prior to the pandemic, was as follows: *S*.*aureus* > *A*.*baumannii* complex > *P*.*aeruginosa* > *K*.*pneumoniae*, while in 2020 and 2021, post-pandemic, the order was as follows: *S*.*aureus* > *P*.*aeruginosa* > *K*.*pneumoniae* > *A*.*baumannii* complex. The respiratory microorganisms showed a clear change in variation after the onset of the COVID-19 pandemic, with a large decrease in the *A. baumannii* complex (Figure 1).

The comparison of the mean number of isolated bacteria from the received respiratory samples before and after the onset of the COVID-19 pandemic showed that the number was 1,780 prepandemic and 1,415 postpandemic, which showed a reduction of 20.5%, and the number of most strains was reduced with the exception of *C. albicans*, *E. aerogenes*, and *P. mirabilis*. The strains showing over 50% reduction in isolation were *S. pneumoniae* (60.5%), *H. influenzae* (67.2%), and *M. catarrhalis* (78.1%).

For *S. aureus* and *K. pneumoniae*, their isolation frequency did not decrease statistically significantly for two years after the declaration of the COVID-19 pandemic. In the case of *C. albicans*, the isolation frequency increased significantly after the declaration of the pandemic (*p* < 0.05) (Figure 1).

### 3.4. Distribution of Respiratory Microorganisms by Age

A comparison of the distribution of the respiratory microbial strains isolated from 2018–2021 by age showed that among the 6,389 cultured bacteria, the largest proportion (1,719; 26.9%) were isolated from the age group in their 70s in the following order: *S. aureus* (*n* = 386, 22.5%), *A. baumannii* complex (*n* = 298, 17.3%), *P. aeruginosa* (*n* = 281, 16.3%), *K. pneumoniae* (*n* = 217, 12.6%), and *C. albicans* (*n* = 99, 5.8%). They were followed in order by the age groups in their 80s (*n* = 1,506; 23.6%), 60s (*n* = 1,320; 20.7%), and 50s (*n* = 856; 13.4%).

**Figure 2:**
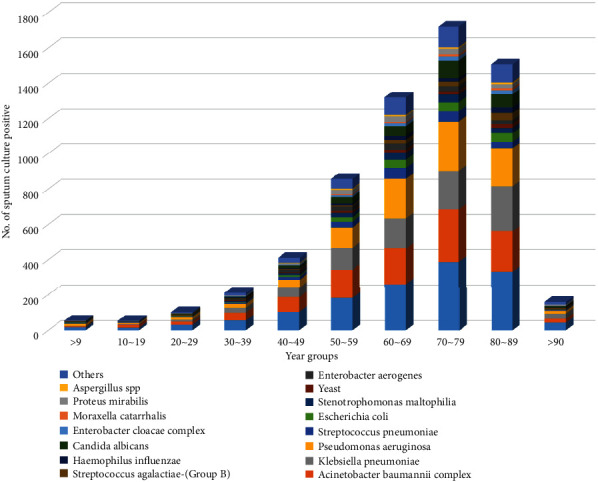
Number of bacteria and fungi identified in sputum, bronchial aspirate, and transtracheal aspirate samples by age groups.

In contrast, the lowest isolation frequency was seen in teenagers (*n* = 53; 0.8%), followed by children aged ≤9 years (*n* = 54; 0.8%), followed in order by the age groups in their 20s (*n* = 102; 1.6%), 90s and above (*n* = 159; 2.5%), 30s (*n* = 210; 3.3%), and 40s (*n* = 410; 6.4%) (Figure 2).

## 4. Discussion

In this study, a retrospective analysis was performed on the data of 15,398 outpatients at a hospital to assess the effects of the changes in personal hygiene practices, such as mask-wearing and hand washing which have been promoted as part of the NPI strategy to manage the COVID-19 pandemic, based on the frequency of respiratory pathogens.

The distribution of each strain of isolated pathogen from the respiratory samples from 2018–2021 showed that the total number of samples was 39,814 out of which the number of positive samples was 12,823, indicating a positive sample rate of 32.2%. Among the 12,823 positive samples, duplicated strains were excluded, but the strains repeatedly isolated in the following year were included so that a total of 6,389 strains were isolated from 4,454 patient samples. The number of respiratory samples showed a trend of annual increase until 2019 before the COVID-19 pandemic, while a reduction of 6.7% was found after the COVID-19 pandemic. This is presumed to be due to the impact of the COVID-19 pandemic on the level of received samples.

By age, the highest frequency of isolation was found for the age group in their 70s, followed by those in their 80s, 60s, and 50s. This may indicate a close correlation with the older adult population in the Chungcheongnam-do region with a notably large difference between the urban and metropolitan areas and the agricultural areas at the accelerated rate of aging [26, 27]. Gender analysis showed that male patients had a higher percentage of isolation than female patients at 64.9% for all the age groups up to their 80s, while for the age group in their 90s, there was a higher percentage for female patients. This is presumed to be due to the gap in life expectancy between men and women in South Korea. In 2020, the life expectancy was greater for women (86.5 years) than for men (80.5 years) [28] and consequently, the population of elderly women is expected to be larger than that of men, resulting in a greater number of female patients in their 90s. The microbial isolation from older adults aged 60 years was 74.0%, among which 45.9% was from male patients and 28.0% was from female patients with a 17.9% higher percentage of isolation from women aged > 60 when compared with men. According to the 2020 data from the Statistics Korea, the mortality due to pneumonia in men and women was 47.2 and 39.5, respectively, per 100,000 persons, indicating a higher rate of mortality in men.

Among the outpatients from 2018–2021, the number of patients positive for pneumonia was 1,313 in 2018 and 1,203 in 2019 before the COVID-19 pandemic and 956 in 2020 and 982 in 2021 after the COVID-19 pandemic. Compared to the time before the pandemic, the number of pneumonia-positive patients showed a reduction of 23.0%. This coincided with the data from the Health Insurance Review and Assessment Service (HIRA) as the number of pneumonia patients showed a gradual increase from 2017 (*n* = 1,319,000) to 2018 (*n* = 1,343,000) to 2019 (1,406,000), while there was a decrease in 2020 (*n* = 670,000) by 52.3% when compared to the previous year [29]. However, such a high rate of reduction was not observed in this study due to the fact that university hospitals generally have a high number of critical patients.

The bacterial strain with the highest frequency of isolation was *S. aureus*, followed by the *A. baumannii* complex, *P. aeruginosa*, *K. pneumoniae*, *C. albicans,* and *S. pneumoniae*. The most frequently isolated strain, the *S. aureus*, is a strain of the resident flora that may cause nosocomial infections and opportunistic infections. Due to these characteristics, the influence of the frequency due to the COVID-19 pandemic was insignificant. The second most frequently isolated strain, the *A. baumannii* complex, is mainly acquired via nosocomial infection and tends to increase in cases of multiple drug resistance (MDR). The rate of reduction compared to the time before COVID-19 was 43.1%, which implied that, improved personal hygiene had an effect on nosocomial infections. *K. pneumoniae* is an opportunistic species that causes hospital-acquired infections, and it has shown indications that it is not affected by the COVID-19 pandemic. For *C. albicans*, since the identification of *C. albicans* is affected by the host condition and not infection through the respiratory route, preventive measures, including improved hygiene, have little impact on their frequency.

In the case of *S. pneumoniae* as the representative strain of community-acquired pneumonia, an increasing trend was observed in 2018 and 2019 before the COVID-19 pandemic, and there was a gradual reduction from 2020, which lead to lead a 72.6% reduction in 2021 when compared to 2019. The results suggest that hand washing and mask-wearing as the main methods of personal hygiene management could substantially reduce the rate of infection by *S. pneumoniae*. In addition, the other known strains of community-acquired pneumonia, *H. influenzae* and *M. catarrhalis*, showed considerable reductions after the COVID-19 pandemic. For *H. influenzae*, the isolation frequency showed an increase by 8.6% in 2019 when compared to 2018, while after the COVID-19 pandemic, the frequency decreased by 47.4% in 2020 when compared to 2019 and by 89.5% in 2021 to show an even greater rate of reduction. For *M. catarrhalis*, 32 cases in total were found before the COVID-19 pandemic, which was reduced to 7 cases, resulting in a 78.1% reduction.

Overall, the improvements in personal hygiene were found to markedly reduce the distribution of the *A. baumannii* complex, *S. pneumonia*, *H. influenzae,* and *M. catarrhalis* after the COVID-19 pandemic, based on the respiratory microorganism analysis before and after the pandemic in South Korea. There was no significant difference in the statistics of nosocomial microbes before and after the COVID-19 pandemic, except in the case of *A. baumannii*. It is thought that the ecosystem of nosocomial infections native to hospitals is not significantly affected by the changes caused by COVID-19. Notably, the strains of community-acquired pneumonia namely, *S. pneumoniae*, *H. influenzae,* and *M. catarrhalis* were shown to have decreased by 60.5%, 67.2%, and 78.1%, respectively, after the COVID-19 pandemic. This indicated a reduction in the community transmission of respiratory pathogens after the execution of public health care strategies and focused on the NPI, and that such strategies were highly effective in controlling respiratory infection. Furthermore, the NPI strategies are anticipated to be a crucial component of the future infectious disease control against respiratory pathogens and to offer additional benefits for general public health care measures.

This study was conducted on the respiratory microorganisms isolated from the samples of patients visiting a university hospital in Chungcheongnam-do, South Korea, from 2018–2021, which posed spatial and temporal constraints. Thus, a more extensive study should be conducted in the future.

## 5. Conclusions

The results of this study suggest that the measures to prevent the spread of COVID-19 in the general population had a notable impact on respiratory microorganisms in general and a prominent effect on the prevention of respiratory infection.

## Figures and Tables

**Table 1:** Age and sex distribution of the study population.

Age group	Male (%)	Female (%)	Total (%)
< 9	25 (0.9)	16 (1.0)	41 (0.9)
10–19	25 (0.9)	11 (0.7)	36 (0.8)
20–29	54 (1.9)	20 (1.3)	74 (1.7)
30–39	92 (3.2)	33 (2.1)	125 (2.8)
40–49	223 (7.7)	70 (4.5)	293 (6.6)
50–59	428 (14.8)	163 (10.4)	591 (13.3)
60–69	622 (21.5)	266 (17.0)	888 (19.9)
70–79	763 (26.4)	449 (28.8)	1,212 (27.2)
80–89	600 (20.8)	462 (29.6)	1,062 (23.8)
> 90	60 (2.1)	72 (4.6)	132 (3.0)
Total	2,892 (100)	1,562 (100)	4,454 (100)

**Table 2:** Number of patients, number of positive patients, number of samples, number of positive samples by year.

	2018	2019	2020	2021	*p* value
Number of patients	4,290	4,298	3,527	3,283	*p* < 0.0001
Number of positive patients	1,313	1,203	956	982	*p* = 0.34
Number of samples	9,961	10,637	9,443	9,773	*p* < 0.0001
Number of positive samples	3,349	3,709	2,771	2,994	*p* < 0.0001

**Table 3:** Number and isolation frequency of bacteria and fungi identified in sputum, bronchial aspirate, and transtracheal aspirate samples by year.

Organism	2018 (%)	2019 (%)	2020 (%)	2021 (%)	Total (%)
*Staphylococcus aureus*	415 (22.8)	384 (22.1)	294 (21.5)	327 (22.4)	1,420 (22.2)
*Acinetobacter baumannii* complex	355 (19.5)	332 (19.1)	184 (13.4)	207 (14.2)	1,078 (16.9)
*Pseudomonas aeruginosa*	214 (11.8)	275 (15.8)	266 (19.4)	199 (13.6)	954 (14.9)
*Klebsiella pneumoniae*	273 (15.0)	205 (11.8)	182 (13.3)	235 (16.1)	895 (14.0)
*Candida albicans*	76 (4.2)	104 (6.0)	104 (7.6)	99 (6.8)	383 (6.0)
*Streptococcus pneumoniae*	65 (3.6)	102 (5.9)	38 (2.8)	28 (1.9)	233 (3.7)
*Escherichia coli*	57 (3.1)	45 (2.6)	32 (2.3)	57 (3.9)	191 (3.0)
*Stenotrophomonas maltophilia*	52 (2.9)	31 (1.8)	38 (2.8)	35 (2.4)	156 (2.4)
*Enterobacter aerogenes*	39 (2.1)	26 (1.5)	23 (1.7)	49 (3.4)	137 (2.1)
*Proteus mirabilis*	19 (1.0)	41 (2.4)	43 (3.1)	27 (1.9)	130 (2.0)
*Streptococcus agalactiae*-(group B)	36 (2.0)	29 (1.7)	29 (2.1)	21 (1.4)	115 (1.8)
*Haemophilus influenzae*	35 (1.9)	38 (2.2)	20 (1.5)	4 (0.3)	97 (1.5)
*Enterobacter cloacae Complex*	30 (1.7)	17 (1.0)	14 (1.0)	24 (1.6)	85 (1.3)
*Moraxella catarrhalis*	23 (1.3)	9 (0.5)	6 (0.4)	1 (0.1)	39 (0.6)
*Aspergillus* spp.	9 (0.5)	13 (0.8)	5 (0.4)	9 (0.6)	36 (0.6)
Others	124 (6.8)	87 (5.0)	91 (6.7)	138 (9.5)	440 (6.9)
Total	1,822 (100)	1,738 (100)	1,369 (100)	1,460 (100)	6,389 (100)

## Data Availability

The data used to support the findings of this study are available from the corresponding author upon request.
